# High throughput synthetic lethality screen reveals a tumorigenic role of adenylate cyclase in fumarate hydratase-deficient cancer cells

**DOI:** 10.1186/1471-2164-15-158

**Published:** 2014-02-25

**Authors:** Michael Boettcher, Andrew Lawson, Viola Ladenburger, Johannes Fredebohm, Jonas Wolf, Jörg D Hoheisel, Christian Frezza, Tomer Shlomi

**Affiliations:** 1 Division of Functional Genome Analysis, Deutsches Krebsforschungszentrum, Heidelberg, Germany; 2 MRC Cancer Unit, Cambridge Biomedical Campus, University of Cambridge, Hutchison/MRC Research Centre, Box 197, Cambridge CB2 0XZ, UK; 3 Lewis-Sigler Institute for Integrative Genomics, Princeton University, Princeton, NJ 08544, USA; 4 Department of Computer Science, Technion, Haifa 32000, Israel

**Keywords:** HLRCC, Fumarate hydratase-deficiency, High-throughput RNAi screen, Adenylate cyclase

## Abstract

**Background:**

Synthetic lethality is an appealing technique for selectively targeting cancer cells which have acquired molecular changes that distinguish them from normal cells. High-throughput RNAi-based screens have been successfully used to identify synthetic lethal pathways with well-characterized tumor suppressors and oncogenes. The recent identification of metabolic tumor suppressors suggests that the concept of synthetic lethality can be applied to selectively target cancer metabolism as well.

**Results:**

Here, we perform a high-throughput RNAi screen to identify synthetic lethal genes with fumarate hydratase (FH), a metabolic tumor suppressor whose loss-of-function has been associated with hereditary leiomyomatosis and renal cell carcinoma (HLRCC). Our unbiased screen identified synthetic lethality between FH and several genes in heme metabolism, in accordance with recent findings. Furthermore, we identified an enrichment of synthetic lethality with adenylate cyclases. The effects were validated in an embryonic kidney cell line (HEK293T) and in HLRCC-patient derived cells (UOK262) via both genetic and pharmacological inhibition. The reliance on adenylate cyclases in FH-deficient cells is consistent with increased cyclic-AMP levels, which may act to regulate cellular energy metabolism.

**Conclusions:**

The identified synthetic lethality of FH with adenylate cyclases suggests a new potential target for treating HLRCC patients.

## Background

Until recently, the identification of synthetic lethal interactions in human cells was based on screens using chemical libraries or on inferred synthetic lethality in model organisms such as *S. cerevisiae*[1]. Recent advances in RNAi technology have enabled systematic screening for synthetic lethal interactions in human cells. This was done either by a traditional forward or backward genetics approach [2]. In forward genetics, gene knockdown effects are measured in multiple, genetically varied cancer cell lines, with synthetic lethality identified based on correlation between a specific genetic change and sensitivity to specific gene knockdown. The backward genetics approach involves the generation of a pair of isogenic cell lines with a specific genetic change and the consequent screening of both cell lines for their response to gene knockdowns. The actual screen for gene knockdown effects is performed either individually for each short hairpin RNA vectors (shRNA), or by pooling them together using molecular barcode techniques [2]. Such approaches have recently been applied to identify synthetic lethal partners with central oncogenes and tumor suppressors, such as p53, RAS, BRCA, and VHL, all major regulators of cancer signaling pathways [3-7].

The recent identification of somatic mutations in cancer that affect metabolic enzymes suggests that the concept of synthetic lethality may be successfully applied to target cancer metabolism [8-11]. Such genetic mutations include the identification of two metabolic tumor suppressors in TCA cycle, fumarate hydratase (FH) and succinate dehydrogenase (SDH), and oncogene, isocitrate dehydrogenase (IDH), as well as other passenger loss-of-function mutations [8-10]. Loss-of-function mutations in FH have been associated with a number of diseases including hereditary leiomyomatosis and renal cell carcinoma (HLRCC), a cancer syndrome characterized by a malignant form of renal cancer [12]. A recent study by Frezza et al. combined experimental metabolomics and computational modeling to identify a metabolic shift that occurs in FH deficient cells, characterized by a truncated TCA cycle and diversion of glutamine-derived carbons into the heme biosynthesis and degradation pathway [11]. Of note, the inhibition of this pathway selectively affected the viability of FH-deficient cells, while sparing the FH-proficient counterpart. While this represents a promising application of metabolic synthetic lethality, no high-throughput screening for synthetic lethality has been performed so far.

Here, we perform a high-throughput screen for genes that are synthetic lethal with FH in a human FH-silenced embryonic kidney cell line (HEK293T), utilizing an RNAi library targeting over 10,000 candidate genes. Our unbiased screen revealed that several genes in the heme metabolism are synthetic lethal with FH, in accordance with previous findings [13]. Furthermore, we identified an enrichment of synthetic lethal interactions with adenylate cyclases. We validated these findings in HEK293T and in FH-deficient HLRCC-patient derived cells (UOK262) via both genetic and pharmacological inhibition and demonstrated that that the FH-deficient cells are characterized by an increased turnover of cAMP.

## Results

### Utilizing pooled RNAi screen to identify synthetic lethality with FH

**Figure 1:**
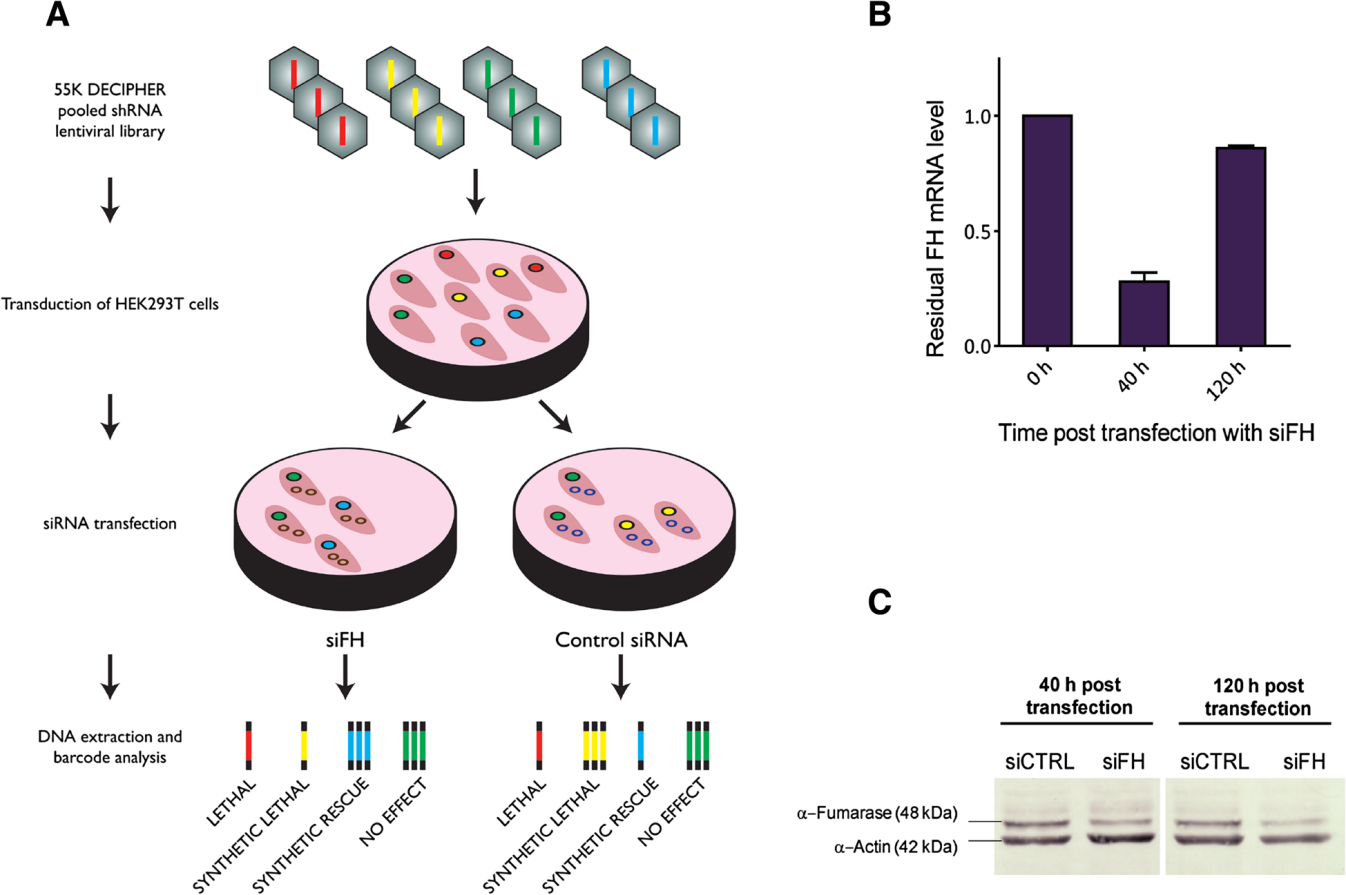
**Schematic of RNAi screen with analysis of fumarate levels throughout the screen. (A)** Schematic of FH synthetic lethality screening strategy. Briefly, the screen involves three steps: (i) cell transduction with a library of shRNA agents; (ii) splitting the culture and silencing FH in one subculture; (iii) quantifying shRNA agent abundance by means of barcode sequencing. The expression of different shRNAs can have lethal (red), synthetic lethal (yellow), synthetic rescue (blue), or no effect (green) on the proliferation of each cell during the screen. **(B)** Residual FH mRNA levels at indicated times post siRNA transfection. **(C)** Residual FH protein level at indicated times post siRNA transfection.

To identify genes that are synthetic lethal with FH, we applied an approach that combines the pooled shRNA silencing of 10,000 different genes with siRNA silencing of FH in a FH-proficient embryonic kidney cell-line HEK293T (Figure 1A). HEK293T cells were transduced with a library of shRNA agents with an average of 5 agents per gene (lentiviral 55 K Decipher shRNA expression library. Cellecta, Mountain View, USA). The complete set of genes targeted by the Decipher library is shown in Additional file 1. Transduction with low viral titer (MOI = 0.3) ensured a maximum of one viral integration event, and hence one gene knockdown per target cell. Seven days post-transduction, cells were sub-cultured under two different conditions: One fraction of cells was transfected with siRNA targeting the expression of FH and another fraction was transfected with a non-targeting control siRNA. The mRNA levels of FH were initially reduced to below 30 percent and recovered almost completely at 120 h post transfection (Figure 1B). On the other hand, levels of Fumarase, the protein encoded by FH, were found to be reduced for up to 120 h post transfection (Figure 1C). The reduction of Fumarase to levels even lower than the ones shown in Figure 1C lead to cell death (unpublished data), since Fumarase is essential for the survival of HEK293T cells. Consequently, we tried to avoid such low Fumarase levels during the screen. Finally, cells were harvested and each of the 55 K expression constructs was quantified via next-generation sequencing of associated molecular barcode tags. Cells that expressed a synthetic lethal shRNA with FH would consequently be depleted in the siFH pool compared to siCTRL pool (Figure 1A). We then computed the ratio of abundances of each shRNA between the siFH the siCTRL and pools. A complete list of shRNA expression constructs together with the corresponding abundance ratios is shown in Additional file 2. We consider a gene as a candidate to be synthetic lethal with FH if the ratio of abundances for at least half of the shRNA agents targeting the gene is lower than a threshold. In order to account for screen specific ‘noise’ levels, this threshold was computed from the standard deviation of abundance ratios obtained from twenty-one shRNA agents targeting a negative control (Luciferase). Out of the total 10,455 genes analyzed, 340 candidate synthetic lethal genes were found (Additional file 3).

Next, we searched for pathways that are significantly enriched with candidate FH-synthetic lethal genes. Interestingly, we found 7 KEGG and Reactome enriched pathways (Table 1). Among these pathways, heme metabolism and in particular HMOX2, FECH, UGT1A3, UGT2A3 were found to be synthetic lethal with FH, in striking accordance with previous observations by Frezza et al. [11]. Another pathway enriched with candidate synthetic lethal genes with FH is the ‘metabolism of carbohydrates’ pathway, consisting of glucose transporter SLC2A1 and three glycolytic enzyme coding genes, TALDO1, ENO3, PKLR. This pathway enrichment is consistent with an increase in glucose uptake and glycolytic flux due to FH deletion, as has previously been demonstrated [11,14]. The pathway showing the highest enrichment of synthetic lethal genes is ‘GABA B receptor activation’, consisting of four adenylate cyclase genes ADCY3, ADCY6, ADCY7 and ADCY9 (out of a total of ten known adenylate cyclase genes). Given these surprising findings, we decided to focus on validating the synthetic lethality with adenylate cyclase and explore the effect of FH deletion on cyclic-AMP level.

**Table 1:** REACTOME and KEGG pathways enriched with candidate FH-synthetic lethal genes

**Pathway**	**Genes**	***p*-value**
REACTOME_GABA_B_RECEPTOR_ACTIVATION	ADCY3, ADCY6, ADCY7, ADCY9, GNG3, KCNJ4, GNG4	0.0001
KEGG_CYTOKINE_CYTOKINE_RECEPTOR_INTERACTION	BMPR1B, CCL22, CSF3, CXCR6, EGF, GH1, IL1RAP, IL22, INHBB, LTA, TNFRSF10D, TNFRSF11A, TNFRSF1B, TNFSF13B, TNFSF8, EDA2R, TNFSF15	0.0024
REACTOME_G_BETA_GAMMA_SIGNALLING_THROUGH_PI3KGAMMA	GNG13, GNG3, PIK3CG, GNG4	0.0035
KEGG_APOPTOSIS	BCL2L1, CASP6, IL1RAP, IRAK1, MAP3K14, PIK3CG, PRKX, TNFRSF10D	0.0072
REACTOME_MHC_CLASS_II_ANTIGEN_PRESENTATION	ARF1, DNM2, OSBPL1A, CTSC, KIF20A	0.0303
KEGG_PORPHYRIN_AND_CHLOROPHYLL_METABOLISM	FECH, HMOX2, UGT1A3, UGT2A3	0.0432
REACTOME_METABOLISM_OF_CARBOHYDRATES	ABCC5, B4GALT3, ENO3, KERA, PC, PKLR, PYGM, SLC2A1, BCAN, NUP205, SLC25A12, TALDO1	0.0453

### Genetic validation of synthetic lethality between adenylate cyclase and FH in the embryonic kidney cell line, HEK293T

**Figure 2:**
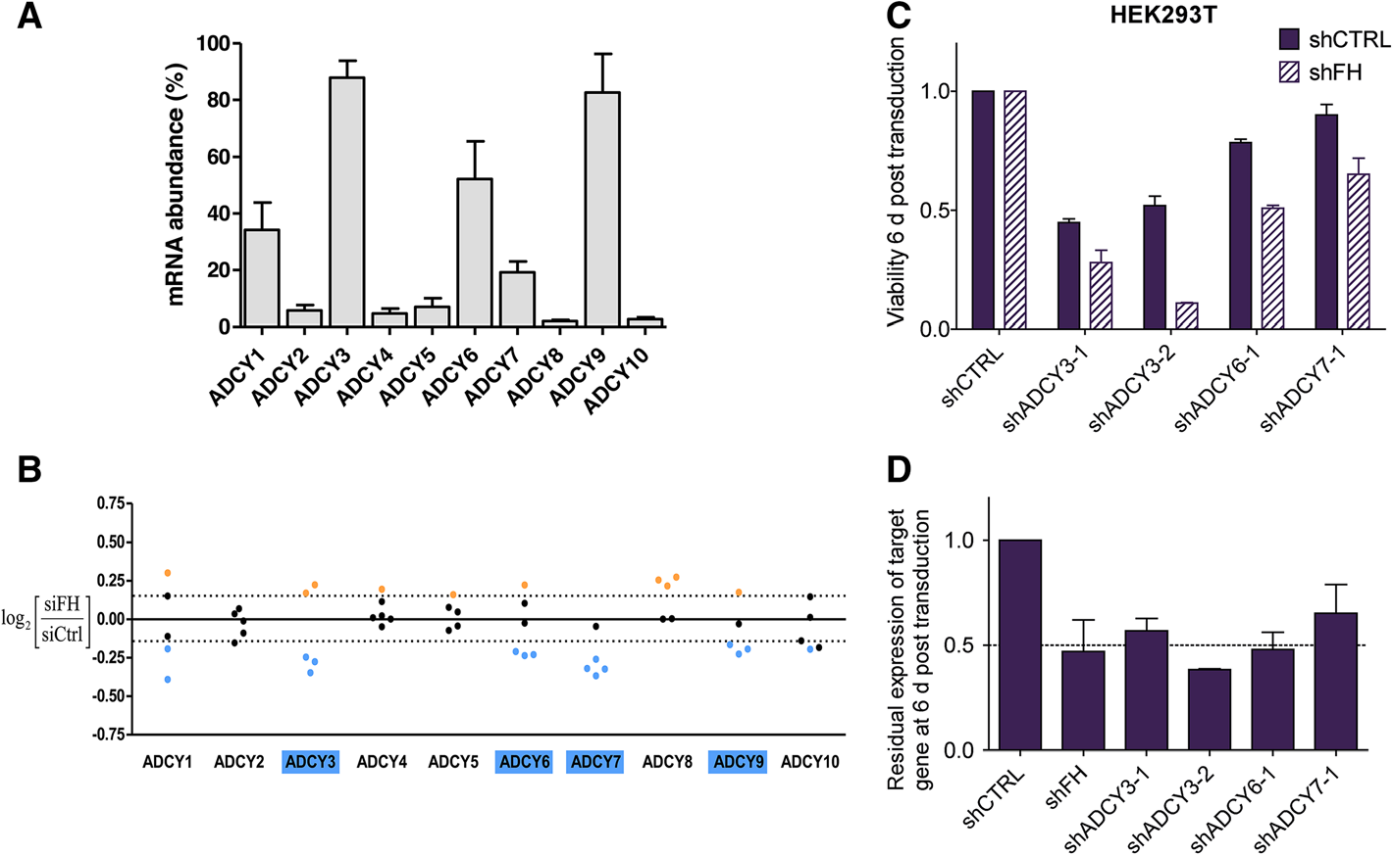
**Adenylate cyclases are synthetic lethal with FH in HEK293T cells. (A)** Expression levels of adenylate cyclase genes in HEK293T cells. **(B)** Ratios of shRNAs targeting all ten known adenylate cyclase genes, as determined from the primary screen. Ratios indicate the abundance of a certain shRNA under siFH vs siCTRL conditions. Negative values indicate depletion, positive values indicate enrichment. Dotted line indicates threshold defined by the distribution of twenty-one negative control shRNAs in order to distinguish background noise from real hits. For genes marked in blue, at least half of the shRNA agents targeting them were depleted below threshold and hence these genes were considered synthetic lethal with FH. shRNA dot colors: yellow: synthetic rescue, black: no synthetic effect, blue: synthetic lethal. **(C)** Viability of FH-impaired HEK293T cells (shFH) and HEK293T expressing endogenous levels of FH (shCTRL) determined after 6 days of expression of indicated shRNA. HEK293T cells were co-transduced with shFH and shADCY shRNA expression constructs or controls, respectively. **(D)** Residual mRNA level of targeted genes after 6 days of expression of indicated shRNA.

To gain insight into why FH-synthetic lethality was found specifically with ADCY3, ADCY6, ADCY7 and ADCY9 among the 10 known adenylate cyclase genes, we examined adenylate cyclase expression in HEK293T. Interestingly, we found that these four genes have the highest expression level among all adenylate cyclases, with the exception of ADCY1 (Figure 2A). Of note, we observed that 2 out of 5 shRNA constructs targeting ADCY1 actually do show a synthetic lethal effect with FH, while the abundance of a third shRNA agent is borderline significant (Figure 2B). Overall, these results suggest that the silencing of the most expressed adenylate cyclase, irrespective of their specific isoform, strongly affects the survival of FH-deficient cells.

To confirm the synthetic lethality between adenylate cyclases and FH, the two highest ranking shRNAs identified by the primary screen, targeting the expression of ADCY3, ADCY6, ADCY7 or ADCY9, were individually sub-cloned into the library expression vector pRSI9 (shRNA sequences are shown in Additional file 4). Cells were co-transduced with lentiviruses containing shADCY expression constructs as well as a shRNA expression construct targeting FH. At 6 days post transduction, expression levels of FH as well as of adenylate cyclases, and cell viability of the cells were determined relative to control cells. Two shRNAs targeting the expression of ADCY3 as well as one shRNA targeting each ADCY6 and ADCY7 were confirmed to be synthetic lethal with FH in HEK293T cells (Figure 2C). In all cases, the shRNA targeting led to approximately 50 percent reduction in gene expression levels (Figure 2D).

### Genetic and pharmacological validation of synthetic lethality between adenylate cyclases and FH in the HLRCC-patient derived cell line UOK262

**Figure 3:**
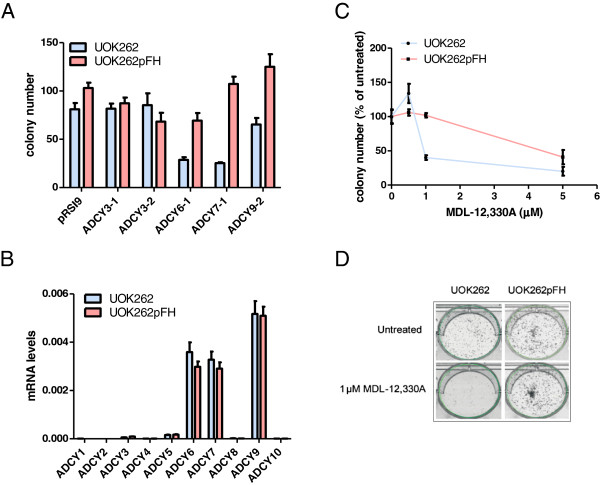
**Inhibition of adenylate cyclases is more toxic to FH-deficient than to FH-expressing HLRCC cells. (A)** Colony formation in UOK262 versus UOK262pFH after the infection with the indicated shRNA. **(B)** Gene expression level of adenylate cyclase genes in UOK262 and UOK262pFH. **(C)** Colony formation following treatment of UOK262 and UOK262pFH cells with indicated concentrations of the adenylate cyclases inhibitor MDL-12,330A. **(D)** Representative images of a colony survival assay of UOK262 and UOK262pFH after the treatment with 1 μM MDL-12,330A for 7 days.

To further validate synthetic lethality between adenylate cyclase and FH, we used tumor cells harboring a germline mutation in FH, the UOK262 renal tumor cells, a metastatic cell line derived from an HLRCC patient [15]. Of note, these cells are devoid of any FH activity and show similar metabolic features to Fh1-deficient mouse cells [13]. Specifically, we silenced ADCY3, ADCY6, ADCY7 and ADCY9 expression and measured colony formation both in UOK262 and in an isogenic cell line in which FH was reintroduced (UOK262pFH) [11]. Of note, we found that ADCY6, ADCY7 and ADCY9, but not ADCY3 were synthetic lethal with FH (Figure 3A). Since these results are in partial agreement with our findings in FH-deficient HEK293T cells, we analyzed adenylate cyclase expression data measured by qPCR in UOK cells. Interestingly, we found that ADCY6, ADCY7, and ADCY9 are the most abundant adenylate cyclase in UOK262 cells, while ADCY3 is very poorly expressed in these cells. These results strongly suggest that targeting the most abundant adenylate cyclases is again sufficient to affect cell viability of FH-deficient cells (Figure 3B).

Next, we wanted to confirm synthetic lethality between adenylate cyclases and FH using a pharmacological approach. To this end, we tested the effects of the adenylate cyclase inhibitor MDL-12,330A on the UOK262 cell lines. Both cell lines were treated with increasing concentrations of 0.5, 1 and 5 μM MDL-12,330A for 7 days, after which colonies were counted (Figure 3C). It was found that UOK262 cells were more sensitive to this adenylate cyclase inhibitor than their FH reconstituted UOK262pFH counterparts. Particularly, at a concentration of 1 μM MDL-12,330A we found that UOK262 cells formed less than half as many colonies as UOK262pFH cells (Figure 3C and D).

### Cyclic-AMP production is increased following FH deletion and supported by a drop in nucleotide phosphodiester expression

**Figure 4:**
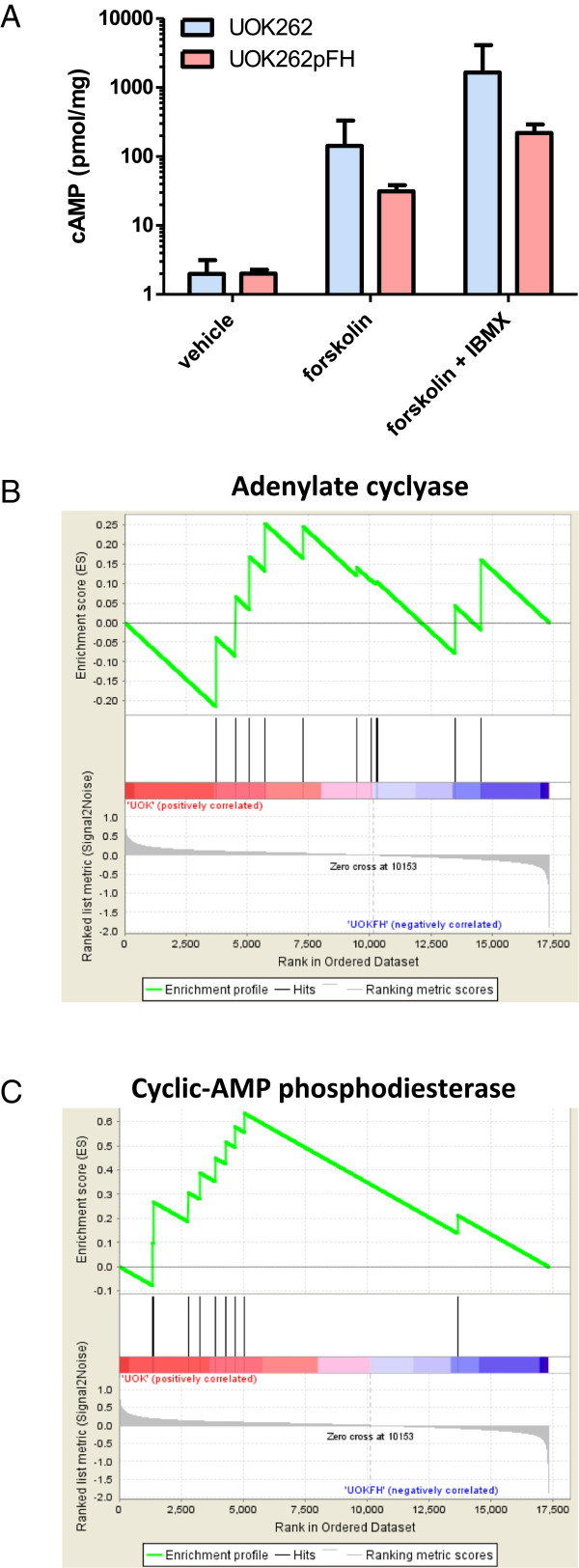
**Increase in cyclic-AMP production following FH deletion. (A)** Cyclic-AMP level in UOK262 versus UOK262pFH following treatment with the adenylate cyclase activator forskolin alone or in combination with PDEs inhibitor IBMX. **(B-C)** Gene set enrichment analysis (GSEA) of changes in expression between UOK262 versus UOK262pFH. Specifically, the x-axis represents an ordering of genes based on their drop in expression following FH deletion. The y-axis in the upper graph represents the enrichment of increasing sets of differentially expressed genes with adenylate cyclases **(B)** or PDEs **(C)**. As shown, the expression of adenylate cyclases does not change significantly following FH deletion **(B)**, while the expression of PDEs significantly drops following FH deletion **(C)**.

The synthetic lethality between adenylate cyclase and FH suggests that cyclic-AMP (cAMP) mediated signaling pathways may play an important role in the survival of FH-deficient cells. Therefore, we investigated the possibility that cAMP homeostasis is deregulated in these cells by measuring cAMP levels in UOK262. Our analysis revealed that steady-state levels of cAMP are maintained very low in both FH-deficient and proficient cell lines (Figure 4A). Nevertheless, when cells were stimulated with the adenylate cyclase activator forskolin alone or in combination with the pan-inhibitor of phosphodiesterases (PDEs) IBMX, the capacity for cyclic-AMP production of UOK262 cells was higher than that of UOK262pFH, suggesting a higher cAMP turnover in FH-deficient cells. Notably though, these results may be biased due to the existence of distinct cAMP pools of mitochondria and cytoplasm [16].

To examine the mechanism that leads to an increased cAMP turnover in the FH deficient cells, we analyzed the expression of adenylate cyclase and cAMP phosphodiesterase (PDE) genes in UOK262 and UOK262pFH cells using Gene Set Enrichment Analysis (GSEA) [17]. We found that while there is no significant change in adenylate cyclase gene expression following FH deletion, the expression of cAMP phosphodiesterase genes significantly drops following FH deletion (Figure 4B-C). To further evaluate the potential importance of cAMP phosphodiesterase for increasing cAMP production, we reexamined the effect of PDEs knockdowns in the synthetic lethality screen. Importantly, we found that the knockdown of 2 out of 9 cAMP phosphodiesterase (PDE1B and PDE7A) showed a synthetic rescue effect, representing a significant enrichment of PDEs as FH-synthetic rescue genes (hypergeometric *p*-value = 0.047). Hence, the knockdown of either PDE1B or PDE7A leads to a higher growth rate when FH is silenced. Synthetic rescue is defined analogously to synthetic lethality, requiring that the ratio of abundances in the siFH versus the siCTRL pools of at least half of the shRNA agents targeting the gene are significantly high (Figure 1A; Methods).

## Discussion

In this study we performed a high-throughput screen for identifying synthetic lethal genes with the tumor suppressor FH, whose loss of function is associated with the development of renal cancer, with the aim of finding novel therapeutic targets for this deadly disease. The method was applied on the FH-proficient embryonic kidney cell line, HEK293T, and involved the double knockdown of genes via a library of shRNA constructs and by means of the direct silencing of FH via siRNA. Identified synthetic lethality in HEK293T between FH and adenylate cyclases was shown to be transferable to FH-deficient HLRCC-patient derived cells (UOK262). Notably, the screen was not carried out in UOK262 cells, which represent a more direct model of HLRCC, because UOK262 and UOK262pFH substantially differ in terms of proliferation speed, transducibility, knockdown efficiency, and knockdown kinetics, which would have influenced the interpretation of the screen results. Instead, HEK293T is a more appropriate cell line for such screens since it can be easily and effectively transfected and transduced and shows negligible proliferation defects upon acute, partial silencing of FH. Inducing a stronger and more permanent knockdown or even knockout of FH might have yielded different screening results. However, this approach would have required the identification of a FH-proficient cell line that tolerates such strong reduction of FH levels for the duration of the screen (over two weeks). Moreover, the partial reduction of FH levels used for this screen, which resulted in only mild proliferation defects in HEK293T cells, allowed us to analyze synthetic lethal interactions over a large dynamic range. In other words, if strong inhibition of FH by itself would result in severely reduced viability, it would be hard to identify genes whose inhibition in combination with FH reduces viability even further, as required for the identification of synthetic lethal interactions.

Besides confirming the crucial role of the heme biosynthesis pathway in FH-deficient cells, this screen revealed an unexpected result, i.e. synthetic lethality with cAMP signaling pathway. These findings prompted us to further investigate the link between FH and cAMP biosynthesis. Although this link has never been established before in eukaryotes, FH expression is closely associated with cAMP signaling in *E. Coli*, indicating an interesting crosstalk between these two pathways [18]. Our results suggest that FH-deficient cells have a higher turnover of cAMP, which might underline the addiction to cAMP-activated protein kinases (PKA)-associated oncogenic pathways. Indeed, PKAs are known to be involved in the regulation of cell proliferation and have previously been shown to participate in the onset and progression of various cancers [19]. Furthermore, cAMP has previously been shown to induce HO-1, the limiting step of the heme biosynthesis and degradation pathway [20]. Therefore, it can be speculated that increased cAMP could also serve to support the expression of HO-1 [21], which is essential for the survival of FH deficient cells [13], as also found in our synthetic lethality screen. Another hypothesis is that FH deficiency lowers oxidative TCA cycle activity and hence CO_2_ and bicarbonate levels, while bicarbonate is known to activate specific adenylate cyclases [22,23]. The down-regulation of bicarbonate-activated adenylate cycles may potentially render the activity of other adenylate cyclases essential for survival. This hypothesis regarding lower bicarbonate levels in FH deficient cells is supported by a recent study showing increased dependence of FH deficient cells on reductive carboxylation of glutamine-derived alpha-ketoglutarate via isocitrate dehydrogenase [24].

## Conclusion

Our results suggest adenylate cyclase as a new potential target for treating HLRCC patients, while the specific mechanism which renders it essential for the survival of FH-deficient cells remains an open question.

## Methods

### Cell culture conditions

HEK293T cells were cultured in DMEM (Invitrogen #41965) supplemented with 10% FCS (Invitrogen #10500) and 1% penicillin/streptomycin 10,000 U (Invitrogen #15140). UOK-262 and UOK-262-FH cells were cultured in DMEM (Invitrogen #21969) supplemented with 2 mM L-glutamine and 10% FCS. Cells were cultured at 37°C in a 5% CO_2_ atmosphere. All cell lines were tested via “Multiplex cell Contamination Test” as previously described [25].

### Packaging of lentiviral pool

DECIPHER library human Module 1 (#DHPAC-M1-P) and human Module 2 (DHDAC-M2-P) were obtained from Cellecta Inc. (Mountain View, U.S.). For packaging into lentiviral particles, plasmid DNA from Modules 1 and 2 was co-transfected into HEK293T cells together with the second generation helper plasmids psPAX2 and pMD2.G. One day before transfection, HEK293T cells were seeded into nine 175 cm^2^ flasks (Greiner Bio One #660175) at 7.0E + 04 cells per cm^2^ in standard DMEM medium with freshly added L-Glutamine (2 mM final) and without penicillin/streptomycin. At the day of transfection, 60 μg from each Module was mixed with 100 μg pMD2.G, 200 μg psPAX2 and 600 μl PLUS™ Reagent (Invitrogen #11514-015) into a total volume of 13 ml Opti-MEM® (Invitrogen #11058-021). The plasmid mix was incubated for 15 min at RT before mixing with 14 ml Opti-MEM® containing 900 μl Lipofectamine™ (Invitrogen #18324020). The complete transfection mix was incubated for another 15 min at RT before adding 2.9 ml to each cell culture flask containing HEK293T cells. At 24 h post transfection the medium in each culture flask was replaced by 20 ml high serum DMEM (30% FCS) containing DNase (1 U/ml, QIAGEN #19101) and MgCl_2_ (4 mM). Addition of DNase helps reducing carryover of plasmid DNA into the viral pool. At 24 h after addition of high serum medium, the lentivirus containing supernatant was harvested and stored at 4°C. To each culture flask 20 ml fresh high serum medium were added and flasks were incubated for an additional 24 h. Finally, the complete lentiviral harvest was pooled to a total volume of 360 ml and passed through a 0.45 μm PES filter (Nalgene #166-0045). Lentiviral pools were stored in aliquots at −80°C for further use.

### Pooled RNAi screen

HEK293T cells were seeded 24 h prior to transduction. A total of 5.0E + 07 cells were seeded for transduction with each Module into 25 cell culture flasks the size of 175 cm^2^. Cells were transduced with the lentivirally packaged Modules at low multiplicity of infection (MOI = 0.3) in standard DMEM containing 3.5 μg/ml Polybrene® (Sigma-Aldrich #107689). As a result, we anticipate that each of the 27,500 shRNA expression plasmids present in each Module integrated into the genome of 540 individual cells. The viral supernatant was replaced 24 h later with standard DMEM culture medium containing 2 μg/ml cell culture tested puromycin dihydrochloride (Sigma-Aldrich #5858-2). Selection was continued for an additional 5 days after which cells were transferred into fresh standard DMEM medium without antibiotics and grown for an additional 24 h. Following this, 2.8E + 07 cells transduced with either Module were transferred into four new 175 cm^2^ flasks in standard DMEM without antibiotics and freshly added L-Glutamine (2 mM). Cells in two flasks were transfected with a mix of two siRNAs targeting the expression of Fumarate Hydratase (siFH) while cell in the two additional flasks were transfected with of an AllStars negative control siRNA (siCTRL). For siFH transfection, 18.5 ml Opti-MEM® containing 1 ml HiPerfect (QIAGEN #301707) were mixed with 220 μl siFH_2 (20 μM QIAGEN #SI00002989) and 220 μl siFH_3 (20 μM QIAGEN #SI00002996). For the siCTRL transfection, 440 μl AllStars negative control siRNA (20 μM QIAGEN #1027281) was mixed with identical amounts of Opti-MEM® and HiPerfect. Both master mixes were mixed by vortexing and incubated at RT for 10 min. Finally transfection complexes were added dropwise to two culture flasks from each Module. At 40 h post-transfection, cell from each of the four samples (Module1/siFH, Module1/siCTRL, Module2/siFH, and Module2/siCTRL) were transferred into 20 cell culture flasks (175 cm^2^) in standard DMEM medium at 7,0E + 05 cells per cm^2^ and grown for an additional 80 h. By the end of the screen, cells were harvested and cell pellets from each sample were stored in three aliquots (~4.0E + 07 cells per aliquot) at −20°C for further analysis.

### Barcode quantification

Cell pellets from each sample were thawed and resuspended in 5 ml buffer P1 (QIAGEN #19051) supplemented with 100 μg/ml RNase A (QIAGEN #19101) and 0.5% SDS. After 5 min incubation at RT the DNA was sheared sonicating the cells for 5 sec. Following sonication genomic DNA was extracted from each sample using the DNeasy Blood and Tissue Kit (QIAGEN #69504). Total DNA yield was around 300 μg from each sample. PCR amplification of barcodes was carried out in two separate rounds using the Titanium® *Taq* PCR Kit (Clonetech #639211). The reaction composition for the first round PCR was 10 μl 10× Titanium Taq PCR Buffer, 8 μl dNTPs (2.5 mM each), 1 μl Titanium Taq, 3 μl FwdHTS Primer (10 μM), 3 μl RevHTS Primer (10 μM) and 50 μg template genomic DNA adjusted to a total of 100 μl with PCR-grade water. Four reactions were prepared from each sample resulting in a total of 200 μg genomic DNA being used for barcode amplification. The PCR program for the first round PCR was (1) 94°C for 3 min, (2) 94°C for 30 sec, (3) 65°C for 10 sec, (4) 72°C for 20 sec, (5) goto (2) 15 times, (6) 68°C for 2 min, (7) 10°C forever. For each sample, the four first round reactions were pooled and 2 μl of this pool were used as template for second round PCR. In addition to the 2 μl template from the first round PCR, the reaction composition of the second round PCR was 10 μl 10× Titanium Taq PCR Buffer, 8 μl dNTPs (2.5 mM each), 1 μl Titanium Taq, 5 μl FwdGEX Primer (10 μM), 5 μl RevGEX Primer (10 μM) adjusted to a total of 100 μl with PCR-grade water. The PCR program for the first round PCR was (1) 94°C for 3 min, (2) 94°C for 30 sec, (3) 65°C for 10 sec, (4) 72°C for 10 sec, (5) goto (2) 9 times, (6) 68°C for 2 min, (7) 10°C forever. For each sample three second round PCRs were performed and every reaction was purified individually using QIAquick PCR purification Kit (QIAGEN #28106) and eluted in 50 μl elution buffer (EB). Eluted PCR products were analyzed individually via gel electrophoresis and Nanodrop ND1000. PCR products were 106 base pairs long for every reaction and amounts were almost identical. Triplicate reactions from each sample were pooled and purified via gel electrophoresis (3.5% agarose) using QIAquick Gel Extraction Kit (QIAGEN #28704). Gel excision was performed without directly staining the 106 bp band with ethidium bromide but smaller bands which were run alongside with the band for excision. After gel purification the PCR products were eluted in 2× 10 μl elution buffer, heat denatured at 95°C for 5 min and placed on ice. The gel purified fragments were adjusted to equal concentrations (50 ng/μl). Finally 2 μl of each fragment were analyzed on a 3.5% agarose gel and found to be of correct size. Following successful recovery, the barcodes were quantified using a Genome Analyzer IIx (Illumina) and the GexSeqN sequencing primer at a final concentration of 500 nM. Barcodes from Module 1 and Module 2 were sequenced combined in one lane for siFH or siCTRL respectively. In average 3.0E + 07 reads were sequenced per lane. The sequences for all primers used are shown in Additional file 5.

### Analysis of sequencing data

For quantification of read counts per barcode, the Barcode Deconvoluter software was used which is available for download from the Cellecta website. Read counts of individual barcodes were normalized to the average read count in each lane to adjust for varying total read counts in different sequencing lanes. Barcodes with less than 100 reads in the control lane were excluded from analysis (Module 1 = 2.3%; Module 2 = 2.7%). From the remaining barcodes the ratios between read counts after FH knockdown and negative control were calculated. All log_2_ ratios used for further analysis are shown in Additional file 2. Each module included twenty-one negative control shRNA expression constructs targeting the gene Luciferase (Luc) for knockdown. The mean standard deviation from those constructs was calculated as an estimate of variance in the screen. Standard deviations were 0.1595 and 0.1863 for Modules 1 and 2, respectively. Expression constructs with log_2_ ratios lower than the negative standard deviation of Luciferase constructs were considered to be specifically depleted in the FH knockdown sample. The number of constructs per gene that passed those filter criteria was counted. Genes represented by more than half of the constructs they were targeted by, were considered as potentially synthetic lethal interaction partners of FH. In total 340 genes (Additional file 3) out of the 10,455 genes targeted by both Modules (Additional file 1) were considered as candidate genes and used for signaling pathway analysis.

### Pathway enrichment analysis of candidate synthetic lethal genes

A hypergeometric test was used to compute an enrichment *p*-value for 861 KEGG and Reactome pathways. P-values were FDR corrected for multiple testing. Significantly overlapping pathways were filtered by iteratively going over the list of pathways (sorted by FDR corrected *p*-value), removing pathways with more than 50% overlap with previous pathways in the list.

### Cloning of shRNA expression constructs

Candidate oligonucleotide sequences were synthesized and desalted (Sigma). Sequences are shown in Additional file 4. At 5′ the guide strand was synthesized with an additional ACCG overhang and the passanger strand with CGAA to allow subsequent cloning into the BpiI (Fermentas #ER1011) digested pRSI9 vector. Following phosphorylation (T4 PNK, Fermentas #EK0031) and annealing of guide and passanger strand (99°C to 4°C at −0.1°C per second) the double stranded oligonucleotides were ligated into BpiI digested pRSI9 vector with T4 ligase (Fermentas #EL0011) for 1 h at RT. Ligated plasmids were transformed into chemically competent E. coli (strain STBL3) using standard heat shock protocol. Transformed E. coli were transferred onto agar plates supplemented with 100 μg/ml carbenicillin and incubated at 37°C overnight. Single clones were used to inoculate 5 ml LB (100 μg/ml carbenicillin). After 18 h at 37°C with shaking at 200 rpm, plasmids were isolated using QIAprep Spin Miniprep Kit (QIAGEN #27106). Purified plasmids were checked for correctly inserted sequences via sequencing (GATC) with FwdU6 primer. From E. coli cultures harboring the correct plasmid, glycerol stocks were prepared and stored at −80°C for further use.

### Quantitative real-time PCR

Cells were pelleted and total RNA was extracted using Qiagen RNeasy Mini Kit (QIAGEN #74104). RNA concentration was adjusted to 50 ng/μl. The OneStep RT-PCR Kit (QIAGEN #210210) was used according to instructions in the manual and reactions were prepared in a 384 well format with 50 ng template per well. QIAGEN QuantiTect primer shown in Additional file 5 were used. Reactions were run on run on a LightCycler 480 (Roche).

### Western blotting

Samples were run on a 10% SDS-PAGE gel and blotted on nitrocellulose membrane. Primary antibodies were incubated overnight at 4°C. FH antibody (Sigma #2500433) was diluted 1:10,000 and beta-actin antibody (Sigma #A3853) was diluted 1:5,000. Secondary horseradish peroxidase conjugated antibodies were incubated for 1 h at RT. Anti-goat was diluted 1:5,000 and anti mouse was diluted 1:2,000.

### Viability assays

Cells were seeded into 96 well plates at 600 cells per well and transduced at MOI > 1 in appropriate cell culture medium supplemented with 5 μg/ml polybrene. At 6 days post transduction, cell culture medium was replaced by 50 μl medium containing 20 μg/ml resazurine (Acros Organics #418900010). Following incubation fluorescence intensity was detected (Ex = 544 nm/Em =590 nm).

### Colony formation assay

Colony formation assays were performed using a modified version of the sulforhodamine B colorimetric protocol developed by Vichai & Kirtikara, [26]. 600 cells were plated in triplicate into 6-well plates infected with lentiviral particles against the indicated genes or treated with the indicated doses of adenylate cyclase inhibitor MDL-12,330A. At the end of the treatment cells were allowed to recover for 4 days and then fixed using trichloroacetic acid (TCA) at the final concentration of 3% followed by 1 h incubation at 4°C. The plates were washed four times with water before being allowed to air-dry at room temperature overnight. Colonies were stained by incubation with 1 ml 0.05% (wt/vol) sulforhodamine B solution in 1% (vol/vol) acetic acid for 30 min. Unbound dye was removed and the plate washed four times with 1% (vol/vol) acetic acid. Colonies were counted manually and by a ColCount automated colony counter (Oxford Optronix).

### cAMP ELISA

2 × 10^5^ cells were plated onto 6-well plates and cultured in standard medium for 12 h. The medium was replaced with fresh medium supplemented with drugs at the indicated concentrations or DMSO at the maximal concentration used in the drug treatments. At the end of the incubation period, cells were lysed with 250 μl 0.1% DMSO in 0.1 M HCl for 20 min at room temperature. The lysate was centrifuged at 1,000 × g for 5 min and the supernatant split for protein quantification by BCA assay (20 μl) and cAMP quantification (200 μl). The cAMP levels were quantified using the Direct cAMP ELISA kit (Enzo Life Sciences) following manufacturer’s instructions. Absorbance at 405 nm (A405) was detected using an Infinite 200 PRO plate reader (Tecan). The average A405 from blank wells was subtracted from all other wells and the cAMP concentration of samples was interpolated from a four-parameter logistic curve fitted to cAMP standards before normalising by the total protein concentration in each sample. In each individual experiment, drug treatments were performed in triplicate and the cAMP and protein quantifications for each sample were performed in duplicate.

### Availability of supporting data

The complete set of microarray expression data used for GSEA is publicly available under: http://bioinformatics.picr.man.ac.uk/vice/ExternalReview.vice?k=k8%2FrVCknC8jG9iUcnpRXFZap7vs%3D.

## Abbreviations

HLRCC: Hereditary leiomyomatosis and renal cell carcinoma; FH: Fumarate Hydratase; CTRL: Control; ADCY: Adenylate Cyclase; cAMP: cyclic adenosine monophosphate; shRNA: short-hairpin RNA; siRNA: short-interfering RNA.

## Competing interests

The authors declare that they have no competing interests.

## Authors’ contributions

MB established RNAi screening conditions, carried out and analyzed RNAi screens, identified candidate genes and participated in their validation. AL and CF participated in candidate gene validation and carried out all experiments involving chemical inhibitors. VL participated in candidate gene validation. JF and JW prepared lentiviral pools. MB, JDH, CF and TS provided the original concept of the study and advice on experimental design. MB, CF and TS led the data analysis and drafted the manuscript. All authors read and approved the final manuscript.

## Supplementary Material

Additional file 1 Genes included in Decipher library Modules 1 and 2 with Refseq numbers. Click here for file

Additional file 2 **All shRNA constructs included in Decipher library Modules 1 and 2 with Cellecta_IDs, total barcode read counts per construct as well as computed log**_**2**_** fold change ratios from barcode abundances in siFH/siCTRL conditions.** Click here for file

Additional file 3 Genes identified to act synthetically lethal with FH. Click here for file

Additional file 4 Sequences of shRNAs targeting candidate genes. Click here for file

Additional file 5 Sequences of primers used for quantification of barcode abundance and primers used for qRT-PCR. Click here for file

## References

[B1] Hartwell LH Szankasi P Roberts CJ Murray AW Friend SH Integrating genetic approaches into the discovery of anticancer drugs Science 1997 278 5340 1064 1068 10.1126/science.278.5340.1064 9353181

[B2] Nijman SM Synthetic lethality: general principles, utility and detection using genetic screens in human cells FEBS Lett 2011 585 1 1 6 10.1016/j.febslet.2010.11.024 21094158 PMC3018572

[B3] Luo J Emanuele MJ Li D Creighton CJ Schlabach MR Westbrook TF Wong KK Elledge SJ A genome-wide RNAi screen identifies multiple synthetic lethal interactions with the Ras oncogene Cell 2009 137 5 835 848 10.1016/j.cell.2009.05.006 19490893 PMC2768667

[B4] Barbie DA Tamayo P Boehm JS Kim SY Moody SE Dunn IF Schinzel AC Sandy P Meylan E Scholl C Frohling S Chan EM Sos ML Michel K Mermel C Silver SJ Weir BA Reiling JH Sheng Q Gupta PB Wadlow RC Le H Hoersch S Wittner BS Ramaswamy S Livingston DM Sabatini DM Meyerson M Thomas RK Lander ES Systematic RNA interference reveals that oncogenic KRAS-driven cancers require TBK1 Nature 2009 462 7269 108 112 10.1038/nature08460 19847166 PMC2783335

[B5] Bartz SR Zhang Z Burchard J Imakura M Martin M Palmieri A Needham R Guo J Gordon M Chung N Warrener P Jackson AL Carleton M Oatley M Locco L Santini F Smith T Kunapuli P Ferrer M Strulovici B Friend SH Linsley PS Small interfering RNA screens reveal enhanced cisplatin cytotoxicity in tumor cells having both BRCA network and TP53 disruptions Mol Cell Biol 2006 26 24 9377 9386 10.1128/MCB.01229-06 17000754 PMC1698535

[B6] Berns K Hijmans EM Mullenders J Brummelkamp TR Velds A Heimerikx M Kerkhoven RM Madiredjo M Nijkamp W Weigelt B Agami R Ge W Cavet G Linsley PS Beijersbergen RL Bernards R A large-scale RNAi screen in human cells identifies new components of the p53 pathway Nature 2004 428 6981 431 437 10.1038/nature02371 15042092

[B7] Bommi-Reddy A Almeciga I Sawyer J Geisen C Li W Harlow E Kaelin WG Jr Grueneberg DA Kinase requirements in human cells: III. Altered kinase requirements in VHL−/− cancer cells detected in a pilot synthetic lethal screen Proc Natl Acad Sci USA 2008 105 43 16484 16489 10.1073/pnas.0806574105 18948595 PMC2575446

[B8] Muller FL Colla S Aquilanti E Manzo VE Genovese G Lee J Eisenson D Narurkar R Deng P Nezi L Lee MA Hu B Hu J Sahin E Ong D Fletcher-Sananikone E Ho D Kwong L Brennan C Wang YA Chin L DePinho RA Passenger deletions generate therapeutic vulnerabilities in cancer Nature 2012 488 7411 337 342 10.1038/nature11331 22895339 PMC3712624

[B9] King A Selak MA Gottlieb E Succinate dehydrogenase and fumarate hydratase: linking mitochondrial dysfunction and cancer Oncogene 2006 25 34 4675 4682 10.1038/sj.onc.1209594 16892081

[B10] Yan H Parsons DW Jin G McLendon R Rasheed BA Yuan W Kos I Batinic-Haberle I Jones S Riggins GJ Friedman H Friedman A Reardon D Herndon J Kinzler KW Velculescu VE Vogelstein B Bigner DD IDH1 and IDH2 mutations in gliomas N Engl J Med 2009 360 8 765 773 10.1056/NEJMoa0808710 19228619 PMC2820383

[B11] Frezza C Zheng L Tennant DA Papkovsky DB Hedley BA Kalna G Watson DG Gottlieb E Metabolic profiling of hypoxic cells revealed a catabolic signature required for cell survival PLoS One 2011 6 9 e24411 10.1371/journal.pone.0024411 21912692 PMC3166325

[B12] Tomlinson IP Alam NA Rowan AJ Barclay E Jaeger EE Kelsell D Leigh I Gorman P Lamlum H Rahman S Roylance RR Olpin S Bevan S Barker K Hearle N Houlston RS Kiuru M Lehtonen R Karhu A Vilkki S Laiho P Eklund C Vierimaa O Aittomaki K Hietala M Sistonen P Paetau A Salovaara R Herva R Launonen V Germline mutations in FH predispose to dominantly inherited uterine fibroids, skin leiomyomata and papillary renal cell cancer Nat Genet 2002 30 4 406 410 10.1038/ng849 11865300

[B13] Frezza C Zheng L Folger O Rajagopalan KN MacKenzie ED Jerby L Micaroni M Chaneton B Adam J Hedley A Kalna G Tomlinson IP Pollard PJ Watson DG Deberardinis RJ Shlomi T Ruppin E Gottlieb E Haem oxygenase is synthetically lethal with the tumour suppressor fumarate hydratase Nature 2011 477 7363 225 228 10.1038/nature10363 21849978

[B14] Yang Y Lane AN Ricketts CJ Sourbier C Wei MH Shuch B Pike L Wu M Rouault TA Boros LG Fan TW Linehan WM Metabolic reprogramming for producing energy and reducing power in fumarate hydratase null cells from hereditary leiomyomatosis renal cell carcinoma PLoS One 2013 8 8 e72179 10.1371/journal.pone.0072179 23967283 PMC3744468

[B15] Yang Y Valera VA Padilla-Nash HM Sourbier C Vocke CD Vira MA Abu-Asab MS Bratslavsky G Tsokos M Merino MJ Pinto PA Srinivasan R Ried T Neckers L Linehan WM UOK 262 cell line, fumarate hydratase deficient (FH-/FH-) hereditary leiomyomatosis renal cell carcinoma: in vitro and in vivo model of an aberrant energy metabolic pathway in human cancer Cancer Genet Cytogenet 2010 196 1 45 55 10.1016/j.cancergencyto.2009.08.018 19963135 PMC2827193

[B16] Lefkimmiatis K Leronni D Hofer AM The inner and outer compartments of mitochondria are sites of distinct cAMP/PKA signaling dynamics J Cell Biol 2013 202 3 453 462 10.1083/jcb.201303159 23897891 PMC3734087

[B17] Subramanian A Tamayo P Mootha VK Mukherjee S Ebert BL Gillette MA Paulovich A Pomeroy SL Golub TR Lander ES Mesirov JP Gene set enrichment analysis: a knowledge-based approach for interpreting genome-wide expression profiles Proc Natl Acad Sci USA 2005 102 43 15545 15550 10.1073/pnas.0506580102 16199517 PMC1239896

[B18] Tseng CP Yu CC Lin HH Chang CY Kuo JT Oxygen- and growth rate-dependent regulation of Escherichia coli fumarase (FumA, FumB, and FumC) activity J Bacteriol 2001 183 2 461 467 10.1128/JB.183.2.461-467.2001 11133938 PMC94900

[B19] Stork PJ Schmitt JM Crosstalk between cAMP and MAP kinase signaling in the regulation of cell proliferation Trends Cell Biol 2002 12 6 258 266 10.1016/S0962-8924(02)02294-8 12074885

[B20] Durante W Christodoulides N Cheng K Peyton KJ Sunahara RK Schafer AI cAMP induces heme oxygenase-1 gene expression and carbon monoxide production in vascular smooth muscle Am J Physiol 1997 273 1 Pt 2 H317 H323 9249506 10.1152/ajpheart.1997.273.1.H317

[B21] Ryter SW Alam J Choi AM Heme oxygenase-1/carbon monoxide: from basic science to therapeutic applications Physiol Rev 2006 86 2 583 650 10.1152/physrev.00011.2005 16601269

[B22] Buck J Sinclair ML Schapal L Cann MJ Levin LR Cytosolic adenylyl cyclase defines a unique signaling molecule in mammals Proc Natl Acad Sci USA 1999 96 1 79 84 10.1073/pnas.96.1.79 9874775 PMC15096

[B23] Chen Y Cann MJ Litvin TN Iourgenko V Sinclair ML Levin LR Buck J Soluble adenylyl cyclase as an evolutionarily conserved bicarbonate sensor Science 2000 289 5479 625 628 10.1126/science.289.5479.625 10915626

[B24] Mullen AR Wheaton WW Jin ES Chen PH Sullivan LB Cheng T Yang Y Linehan WM Chandel NS DeBerardinis RJ Reductive carboxylation supports growth in tumour cells with defective mitochondria Nature 2012 481 7381 385 388 22101431 10.1038/nature10642 PMC3262117

[B25] Schmitt M Pawlita M High-throughput detection and multiplex identification of cell contaminations Nucleic Acids Res 2009 37 18 e119 10.1093/nar/gkp581 19589807 PMC2764421

[B26] Vichai V Kirtikara K Sulforhodamine B colorimetric assay for cytotoxicity screening Nat Protoc 2006 1 3 1112 1116 10.1038/nprot.2006.179 17406391

